# Assessment of public health laboratory preparedness and response in WHO South-East Asia region during the COVID-19 pandemic: lessons learned and future directions

**DOI:** 10.1016/j.lansea.2024.100496

**Published:** 2024-10-19

**Authors:** Francis Y. Inbanathan, Pushpa R. Wijesinghe, Dhamari Naidoo, Nilesh Buddha, Edwin Ceniza Salvador, Khanh Kim Le, Sandhya Dhawan, Stuart D. Blacksell

**Affiliations:** a World Health Organization South-East Asian Region Office, Delhi, India; b Mahidol-Oxford Tropical Medicine Research Unit, Faculty of Tropical Medicine, Mahidol University, Bangkok, Thailand; c Centre for Tropical Medicine and Global Health, Nuffield Department of Medicine, University of Oxford, Oxford, UK

**Keywords:** SEAR, COVID-19, Laboratory preparedness, Lessons learned

## Abstract

This Health Policy reviews the preparedness and response of public health laboratories in the WHO South-East Asia Region (SEAR) during the COVID-19 pandemic. Through a scoping review and in-depth interviews with key stakeholders, the study identifies successes, challenges, and lessons learned from available literature and the perspective of senior laboratory leaders. Key themes include human resources, health information systems, diagnostic capacity, public risk communication, biosafety, biosecurity, funding, and laboratory network coordination. The findings provide a comprehensive overview of the adaptive capacities of laboratories, the contextual factors influencing their response, and the implications for future pandemic preparedness. This study demonstrates the resilience and adaptability of diagnostic networks in the face of a pandemic but also emphasises the need for strategic resource allocation, highlighting the importance of flexible and scalable networks in managing public health crises. The success of these deployments highlights the necessity for continual investment and coordination of national, regional, and global resources in diagnostic infrastructure to improve preparedness for future public health crises.

**Search strategy and selection criteria**

Scientific articles were identified through searches of PubMed (https://pubmed.ncbi.nlm.nih.gov) using the following term: (“COVID” OR “SARS CoV-2”) AND (“laboratory” OR “testing” OR “diagnostic” OR “diagnosis”) AND (“Thailand” OR “Myanmar” OR “Timor Leste” OR “Democratic People's Republic of Korea” OR “DPR Korea” OR “North Korea” OR “Sri Lanka” OR “Bangladesh” OR “Maldives” OR “Nepal” OR “Bhutan” OR “India” OR “Indonesia”) OR (“pandemic preparedness”). The Google search engine (www.google.com) was used to identify institutional or media reports. Searches were performed for news reports, institutional websites, and peer-reviewed journals, with no defined starting date until March 31, 2023. Only articles published in English were included. We reviewed these articles, and the references cited within for additional relevant reports. Two authors (SDB and KKL) performed searches independently, consulting and cross-checking extracted information as required. Scientific articles, institutional reports and media reports were excluded if they did not relate to laboratories, diagnostic testing, COVID-19 or SARS CoV-2 diagnostics, except those articles describing influenza laboratory pandemic preparedness.

## Introduction

Public health laboratories are vital in disease surveillance, epidemic or pandemic preparedness and response efforts. In January 2020, the WHO declared the COVID-19 outbreak a Public Health Emergency of International Concern (PHEIC) under International Health Regulations. The recommendations from the Emergency Committee emphasised that all countries should be ready for active surveillance and early detection of COVID-19 to prevent the outbreak from spreading further. The 11 member states of the WHO South-East Asia region (SEAR) demonstrated rapid laboratory capacity development for detecting SARS-CoV-2^1^ The COVID-19 pandemic posed significant challenges to entire laboratory systems, which remained essential throughout the pandemic response. The surge phases and peaks of the pandemic placed a considerable burden on laboratory capacity, human resources, and infrastructure. The successful development of laboratory capacities, alongside the challenges encountered in meeting diagnostic demands during the COVID-19 pandemic, underscores the necessity for systematic investigation and documentation of these experiences to inform decision-making for enhancing laboratory preparedness in the region and worldwide. This study uses the results from a scoping review and key informant discussions in WHO SEAR member states to examine pre-pandemic preparedness, the initial surge, the transition to full testing capacity, and the waning response to the pandemic. This information will feed into response efforts and lessons for future epidemic or pandemic preparedness.

We conducted a scoping review and in-depth key informant interviews with SEAR government officials. The scoping review assessed the existing situation, identifying the limitations and challenges in the region. A series of in-depth interviews was conducted with key stakeholders, including laboratory directors, heads of departments, and laboratory managers from public health laboratories in each member state (see Supplementary Table S1 for details). The In-depth interviews were conducted using a semi-structured format, guided by a standardised questionnaire developed based on the scoping review and expert input (see Supplementary Table S2 for details). The study participants and interviewees are listed in Supplementary Table S3. The outcomes of the interviews, including challenges and lessons learned, are summarised in Table 1 and detailed below.

**Table 1:** Summary of challenges and lessons learned following in-depth interviews with WHO South-East Asian Region member state participants.

Human resources	- During the pandemic, the HR capacity was highly lacking in trained staff—especially in the molecular and bioinformatics areas of expertise. - Healthcare workers faced an absurdly high workload, exacerbated by a lack of benefits or support, which could have kept them encouraged and motivated throughout the pandemic. - Staff were often only hired on temporary contracts and trained onsite, so many workers were laid off to adjust to lighter workloads once the pandemic subsided.
Laboratory network coordination	- Member states struggled to coordinate their preparation and response efforts without a laboratory network, compromising their ability to mount an equitable response to the pandemic. - According to the member states, having an established laboratory network from the influenza epidemic contributed significantly to preparedness. - The pandemic disproportionately impacted vulnerable and marginalised populations due to the failure to recognise the importance of coherent, multisectoral laboratory network systems at national and regional levels.
Specimen referral mechanisms and transport systems	- During the pandemic across SEAR, a persistent challenge was delayed sample shipments, transfer, and procurement - Lockdown restrictions played a role in delaying sample transfer - In some nations, it became challenging to communicate through the bureaucracy due to administrative legislation. - The success of a nation's public health emergency response depends on the quality and coverage of its transportation system. Allowing unrestricted movement of personnel, information, and medical supplies are required
Diagnostic capacity	- The pandemic forced a rapid expansion of testing facilities as PCR testing was not routine - Member states should ensure that laboratories at the national and sub-national levels are equipped to test for a wide range of pathogens and that an interconnected diagnostic network is established to relieve pressure on testing facilities in emergency settings - While point-of-care testing and testing facilities in transmission zones are now much more prevalent, challenges remain with the early identification of cases and the collection of sufficient epidemiological data
Public risk communication	- Official communication techniques are critical for educating the public, sharing knowledge about the etiological agent, and offering actionable guidance on how the public should minimise contact and decrease risk. - It is essential to have efficient systems in place that can promptly identify crucial information gaps, produce and modify content, and disseminate it to the general public. - The variables that affect how well the general public receives official communications can be mentioned as public education levels and the population's degree of trust in authority.
Funding and donor support	- National preparedness and response will always be lacking without adequate and timely funding and investment in public health systems. - Insufficient and delayed financing are the primary reasons for the inability to assure global access to life-saving diagnostic tools. Financial resources should be allotted for pandemic preparedness and response to be thoroughly equipped for a future pandemic. - Nations considered fostering open access and transparent scientific cooperation essential, particularly during public health emergencies. - Investments should be made in developing a functional international health information system across SEAR to map and budget economic resources constructively.
Data management systems	- The expanding use of internet-based reporting tools can enhance epidemic and diagnostic reporting, especially in areas with inadequate or porous traditional surveillance systems - Well-funded and robust health information systems can cyclically optimise health system function and resilience to public health emergencies - Routine and emergency health information systems provide real-time, vital crisis data to direct proactive response actions
Biosafety and biosecurity	- Pre-existing risk assessment frameworks are available for individual member states to adapt to their laboratories in spite of remain variances in risk assessment initiatives due to disparities in resources and capacity. - The requirement for biosafety cabinets caused many infrastructural delays across SEAR, especially the time-consuming certification and validation. - The member states' hazardous waste production increased substantially during the COVID-19 pandemic led to significant impact on waste management systems, noticeable and harmful ecology and spread of infection - QA methods were still in development and no validated guidelines had been published made internal QA implementation challenging due to the rely too heavily on conventional methods as on-site auditing and inspections.
People and policies	- The private sector contributed to the considerable success of some member states in the COVID-19 testing programs. - Regulation concerns varied from test and sample validation to monetary regulations: test, sample validation, monetary regulations - Health networks will collapse without efficient mechanisms for planning, management, resource mapping, budgeting and spending, policy formation, coordination, and execution. - For a member state to have good governance, its health decision-making systems and procedures must be accountable, transparent, equitable, and lawfully compliant - Political instability and violence can swiftly undermine institutional capacity and impact surveillance, data management systems, clinical care units, and supply chains
Resource limitations	- Most nations across SEAR struggled to procure reagents, consumables, and other laboratory supplies. Consumables and materials for clinical care equipment were missing, rendering potentially life-saving machinery useless. - PPE shortages and the lack of N95 respirator masks placed the safety of many healthcare workers at risk, specifically those with regular exposure to the virus. - Healthcare personnel worldwide were forced to work without proper protective equipment and were even held responsible for allocating ventilators. - Global efforts and investments should be channeled towards improving surveillance and data management systems to ensure appropriate resource mapping, epidemic-prone disease mapping, and other gaps in resource constraints are prepared for.

SEAR, South-East Asia Region; QA, Quality Assurance.

### Laboratory preparedness and initial response to COVID-19

The scoping review revealed that before the pandemic, the level of preparedness varied significantly across SEAR countries.^2^ Most countries^2^, ^3^, ^4^, ^5^ have invested in robust influenza surveillance systems and pandemic preparedness plans. These pre-existing infrastructures were crucial for their initial response to COVID-19. As a first step, countries utilised their National Influenza Surveillance systems and networks to^5^ facilitate the rapid deployment of SARS-CoV-2 testing capabilities and further expansions.^6^ However, countries with less developed health infrastructures faced significant challenges in expanding testing capacities. The review found that these countries lacked sufficient laboratory capacity and trained personnel, which delayed their ability to respond effectively to the pandemic. The interviews with government officials and laboratory managers echoed these findings, emphasising that countries with established influenza surveillance systems were better equipped to adapt to the demands of COVID-19. Participants highlighted the importance of these pre-existing networks and their crucial role in the rapid scaling of testing capacity. Conversely, some participants discussed their challenges due to their limited pre-pandemic laboratory infrastructure. The interviews also revealed that, despite the varied levels of preparedness, all SEAR member states recognised the potential pandemic threats and took steps to strengthen laboratory capacities, particularly in real-time reverse transcriptase PCR (rRT-PCR) testing, which became a cornerstone of the pandemic response.

### Expansion of diagnostic capacity

The scoping review highlighted that the pandemic necessitated a rapid expansion of diagnostic capacities across the region. Regarding diagnostic technologies, by the end of March 2020, all eleven member states implemented SARS-CoV-2 rRT-PCR diagnostic testing capacity using manual and fully automated high throughput platforms.^7^ Antigen-based rapid diagnostic tests, including cartridge-based GeneXpert®,^8^, ^9^, ^10^ were deployed at field clinics and hospitals to ease the burden on the PCR workload.^11^ Finally, by March 2021, seven member states could perform genome sequencing for SARS-CoV-2, and eight had submitted genetic sequence data to the global platform (GISAID).^12^ Interviews revealed that while rapid antigen tests helped alleviate the burden on PCR facilities, they also introduced challenges regarding quality control and result interpretation. Several countries reported difficulties in ensuring the accuracy and reliability of rapid test results, which were essential for making timely public health decisions.

Laboratory networks expanded rapidly to meet the diagnostic demand for COVID-19 diagnostic testing. For instance, a country with geographical barriers expanded its laboratory network to 685 facilities across 34 provinces^13^^,^^14^ to increase access by utilising all available resources and reducing the need for specimen referrals, significantly enhancing its ability to manage testing demands. Similarly, despite resource limitations, one of the smallest countries in the region managed to establish molecular testing services in all 13 provinces.^8^ This critical expansion allowed over 200,000 molecular tests between March 2020 and February 2022.^8^ However, some countries struggled to meet the diagnostic requirements, merely relying on laboratories concentrated in major urban centres,^15^ which significantly limited access to testing for rural populations.

These included uneven distribution of diagnostic labs, limited qualified staff, implementing guideline challenges, and a shortage of trained personnel.^16^, ^17^, ^18^, ^19^, ^20^ Many countries rapidly deployed additional staff, and some provided proactive training in COVID-19 diagnostic methodologies.^21^ Interviews with laboratory directors and managers provided more profound insights into the challenges associated with this rapid expansion. While the increase in laboratory numbers was a significant achievement, it also exposed critical gaps, particularly in human resources. Many countries reported severe shortages of trained personnel, especially in specialised fields such as molecular biology and bioinformatics. The high turnover of staff, coupled with the reliance on temporary contracts, was a recurring issue. For example, officials from one country discussed the challenges of recruiting and retaining skilled personnel, noting that many workers were hired on short-term contracts and then let go as the initial surge in testing subsided.

### Laboratory network coordination and specimen referral mechanisms

The scoping review emphasised the importance of laboratory network coordination in the pandemic response. Countries with well-established networks could scale up testing more rapidly and efficiently. One middle-income country, for example, reported having more than 1800 laboratories by 2021,^22^ benefiting from pre-existing rapid laboratory support systems. However, coordination challenges were more pronounced in countries with less developed networks. The review noted that these countries faced significant delays in specimen referral and transport, particularly in geographically challenging areas. The interviews provided detailed accounts of these coordination challenges. Participants described how lockdown restrictions and bureaucratic hurdles exacerbated delays in sample transfer, impacting the timeliness of public health responses. In contrast, officials from countries with existing systems highlighted the success of their pre-established laboratory networks, which facilitated faster specimen processing and more effective use of resources. The interviews also revealed that countries with less robust networks had to rely heavily on international reference laboratories for confirmatory testing, further complicating their response efforts.

### Quality assurance programmes

The scoping review identified quality assurance (QA) as a critical component in maintaining the integrity of COVID-19 testing. The WHO's External Quality Assurance Programme (EQAP), implemented in 2020, achieved 100% concordance with national laboratories in ten SEAR member states,^23^ indicating high testing standards. However, the review also pointed out that QA implementation at subnational levels was less consistent, with only 69% of subnational laboratories achieving correct results during EQAP assessments.^24^^,^^25^ This highlighted the need for more robust and widespread QA mechanisms across all levels of the laboratory network. Interviews provided further context to these findings, revealing that many laboratories struggled with implementing QA measures during the pandemic. The reliance on traditional QA methods, such as on-site auditing and inspections, proved problematic due to the restrictions imposed by the pandemic. Officials from large countries discussed the difficulties they faced in maintaining QA standards when in-person inspections were not feasible. The interviews also underscored the importance of developing more flexible QA frameworks that could be adapted to emergency situations, allowing for remote or virtual assessments when necessary.

### Biosafety and biosecurity implementation

Biosafety and biosecurity were identified as significant concerns during the pandemic, particularly as laboratories rapidly expanded their capacities. Several member states invested in adaptive strategies to safeguard staff health while expanding testing capabilities during the pandemic, including implementing strict COVID-19 biosafety measures.^26^^,^^27^ The scoping review highlighted that while some countries had pre-existing risk assessment frameworks, there were considerable disparities in biosafety practices across the region. Many countries recognised enhanced laboratory biosafety requirements,^26^, ^27^, ^28^ and WHO SEAR provided biorisk assessment and management training^29^ and infectious substances shipping training.^30^ The requirement for biosafety cabinets and other protective measures led to infrastructural delays, as certification and validation processes were often time-consuming and difficult to implement under the pressures of the pandemic.

### Finance, external support and cooperation supporting laboratory testing

External assistance and international partnerships were vital in bolstering national responses to the pandemic. Several member states received financial and technical support from various organisations, including WHO, UNOPS, UNICEF, other UN agencies^6^^,^^9^^,^^10^^,^^21^^,^^30^, ^31^, ^32^, ^33^, ^34^ and the Fleming Fund Grant.^8^ Some also had access to the South Asian Association for Regional Cooperation (SAARC) COVID-19 fund.^35^ The interviews also highlighted the importance of international cooperation in addressing these resource limitations. Many countries relied heavily on international donors and organisations for supplies and technical support, which was critical in bridging the gaps in local resources. However, this reliance also exposed vulnerabilities in the sustainability of national public health systems, emphasising the need for more self-reliant approaches to pandemic preparedness.

### Resourcing limitations

Resource limitations were another major theme. The review found that most SEAR countries struggled to procure essential laboratory supplies, such as reagents, consumables, and personal protective equipment (PPE). These shortages were particularly acute during the early stages of the pandemic, limiting the capacity of laboratories to meet the surge in testing demands. Shortages of testing kits, crucial supplies, regulatory hurdles, and financial constraints also impacted various member states.^19^^,^^36^ Regulatory and bureaucratic obstacles in SEAR member states slowed the approval and rollout of new diagnostic tests and technologies, further delaying the pandemic response.^18^^,^^37^^,^^38^ Financial limitations also presented significant obstacles, especially in low-income and middle-income countries (LMICs).^18^^,^^26^ Interviews with laboratory managers and government officials provided a more granular understanding of these challenges. Participants from two countries discussed the difficulties they faced in sourcing and certifying biosafety cabinets, noting that delays in these processes often hindered the expansion of their testing capabilities. Similarly, officials from several countries reported significant challenges in securing PPE for healthcare workers, particularly in the early months of the pandemic. These shortages not only put healthcare workers at risk but also limited the overall effectiveness of the pandemic response.

## Lessons learned

The COVID-19 pandemic has highlighted both the strengths and vulnerabilities of public health laboratory systems across SEAR. This discussion synthesises the key findings from the scoping review and in-depth interviews, offering a comprehensive analysis of the successes, challenges, and strategic recommendations for improving pandemic preparedness and response in the region.

### Successes in laboratory preparedness and response

One of the most notable successes during the pandemic was the ability of several SEAR countries to rapidly scale up their diagnostic capacities. Especially countries that had invested in building robust laboratory networks and influenza surveillance systems prior to the pandemic, were able to adapt these infrastructures to meet the demands of COVID-19 testing swiftly. Rapid expansion from a single national testing centre to multiple sub-national laboratories demonstrates the value of having a well-coordinated and extensive network that can be mobilised in response to a public health crisis.

Similarly, countries with pre-existing rapid laboratory support systems enabled decentralised testing to increase access, highlighting the critical role of preparedness in managing surge capacities. These successes underscore the importance of continuous investment in public health infrastructure, even in times of relative calm, as these investments provide the resilience needed to respond effectively when crises arise. The core pillar of a quality infrastructure program is quality assurance,^39^ overseen by the medical certification and inspection bodies^40^ and controlled by its policymakers and national governments. The WHO's EQAP was instrumental in ensuring the reliability of COVID-19 testing across the region. The high concordance rates achieved by national laboratories participating in EQAP reflect the effectiveness of these international collaborations in maintaining high testing standards during the pandemic; however, the implementation and maintenance were not without challenges. Additionally, international support was crucial in addressing resource gaps, with many countries relying on external funding and technical assistance to procure essential supplies and equipment.

### Challenges and constraints

One of the most pressing issues was the shortage of trained human resources. The rapid expansion of testing capacities put immense pressure on laboratory staff, many of whom were recruited on temporary contracts. While necessary in the short term, this approach was not sustainable in the long run. The high turnover rates and the physical and mental strain experienced by laboratory workers underscore the need for more robust workforce planning and the development of strategies to retain skilled personnel beyond the immediate crisis. The interviews revealed that while some countries could adapt by training new staff or redeploying personnel from other departments, these measures often fell short of the demand. For example, in two countries, the reliance on short-term hiring of staff and the lack of continuity in staffing created significant operational challenges, particularly as the pandemic wore on and the initial wave of recruits left their positions. This situation highlighted the importance of developing more sustainable staffing models that include surge capacity provisions while maintaining a stable core workforce.

Another major challenge was the coordination of laboratory networks, especially in countries with less developed networks. The scoping review and interviews highlighted how the lack of pre-existing networks led to significant delays in testing and an inequitable distribution of diagnostic services. These coordination challenges were exacerbated by logistical issues, such as delays in specimen referral due to geographic barriers and lockdown restrictions. The effectiveness of a public health response is heavily dependent on the ability to quickly and efficiently transport specimens between laboratories, and the delays experienced in some countries compromised the timeliness of their responses. It is also important to recognise that the private sector significantly contributed to COVID-19 testing programs in some countries by setting up private PCR testing labs worldwide. However, regulating the private sector for emergency response was challenging, with concerns about test and sample validation and financial regulations. Some private laboratories dominated diagnostic testing industries without the necessary certifications. It is essential to consider the private sector's role and regulate it based on each country's specific interests for optimal emergency responses.

Resource limitations were another significant constraint, particularly during the early stages of the pandemic.^41^ The scoping review found that many SEAR countries struggled to procure essential laboratory supplies, such as reagents, consumables, and PPE. These shortages were particularly acute in LMICs, where the lack of local production capabilities made them heavily reliant on imports and international donations. The interviews further emphasised that even in countries with relatively robust public health systems, the global competition for supplies created significant challenges, leading to critical shortages that affected the ability of laboratories to meet testing demands. Donors and stakeholders donated machinery often designed for Western standards, rendering it inoperable in specific member states. PPE shortages and lack of N95-type respirators placed healthcare workers at risk, especially those regularly exposed to the virus. Timely global institutional cooperation and technical assistance will be paramount to overcoming resource constraints in outbreaks.

Biosafety and biosecurity were also significant concerns during the pandemic. While some countries had pre-existing risk assessment frameworks, the interviews revealed considerable disparities in regional biosafety practices. The certification and validation processes for critical laboratory equipment, such as biosafety cabinets, were often delayed, leading to bottlenecks in expanding diagnostic capacity. Member states required resources for education and risk management investments.^42^ In several countries, hazardous waste production increased substantially during the pandemic, impacting waste management systems and causing harmful ecological impacts and infection spread.^43^, ^44^, ^45^ Proper disposal of waste products was a problem for member states lacking adequate waste disposal systems.^46^^,^^47^

Maintaining the reliability and integrity of diagnostic testing is essential, particularly during public health emergencies. The pandemic has shown that traditional quality assurance methods, such as on-site inspections, may not always be feasible. The interruption of QA services may jeopardise the operation of clinical care facilities and medical supplies and can severely impact the economic conditions of a nation.^48^ The pandemic clarified that external QA partners relied too heavily on conventional methods, including on-site auditing and inspections. Quality infrastructure was adversely impacted because it was impossible to provide in-person quality assessments given the social distancing safety guidelines.^49^ Although most methods were still in development and no validated guidelines had been published, internal QA implementation was challenging.

Lack of financial support was a critical concern in several SEAR member states. Adequate and timely funding and investment in public health systems are essential for national preparedness and response.^50^ International cooperation has been a cornerstone of the region's response to COVID-19 and will continue to play a vital role in future preparedness efforts. SEAR countries should actively engage in global health networks, sharing data, research findings, and best practices. Collaborative efforts, such as joint training exercises and developing new diagnostic tools, will be essential in building a more resilient and responsive public health system. Countries can better prepare for and respond to emerging health threats by fostering strong international partnerships. Insufficient and delayed financing were primary reasons for the inability to ensure global access to life-saving diagnostic tools.^51^ Financial resources should be allotted for pandemic preparedness and response so that they are thoroughly equipped for future pandemics. This funding should focus on maintaining community-based healthcare capacity by prioritising healthcare workers and essential healthcare services.^52^

### Recommendations for the future

Global support and consensus for pandemic preparedness and response investments are necessary. Following this, preparedness and response capabilities can be extended to primary healthcare at regional levels. Knowledge sharing in research, fostering open access, and transparent scientific cooperation are essential, particularly during public health emergencies. Virtual training among nations facilitated quality assurance, troubleshooting, and maintenance. Developing functional international health information systems across SEAR for economic resource mapping and budgeting is crucial.^53^

Recommendations to address pandemic preparedness challenges in SEAR are presented in Table 2 and are based on and based on lessons learnt from COVID-19^54^ To strengthen public health systems in SEAR, countries must invest in training and retaining skilled laboratory personnel, establishing coherent laboratory networks, and securing sustainable funding mechanisms. Specialised training programs can ensure rapid response during emergencies, while integrated laboratory networks with standardised protocols improve timely access to diagnostics. Training programs for laboratory leadership, such as the Global Laboratory Leadership Programme (GLLP), are critical for building the capacity of current and future leaders in laboratory systems. These programs focus on developing leadership, management, quality assurance, and biosafety skills while fostering collaboration between human, animal, and environmental health sectors.^54^ Governments should prioritise quality assurance programs and enhance biosafety measures through infrastructure upgrades and resilient waste management systems. The WHO Laboratory Biosafety Manual Fourth Edition^55^ and COVID-19-specific guidance^56^ enable the development of standardised locally appropriate biosafety protocols to achieve resilient waste management systems to handle the increased demands during a pandemic. International cooperation is vital for data sharing and joint training efforts, and fostering innovation in diagnostic technologies, including point-of-care testing and genome sequencing, is crucial for future preparedness. Strengthening genomic surveillance capacity is essential for detecting and monitoring pathogens with pandemic potential. This involves establishing regional genomic sequencing networks and ensuring that genomic data is seamlessly integrated into broader public health surveillance systems. Establishing a Regional Diagnostic Advisory Group would provide expert guidance on research and innovation, ensuring effective diagnostic solutions and more robust regional health security. Finally, the pandemic has underscored the importance of access to diverse and adaptable diagnostic tools. Investing in developing point-of-care testing technologies and expanding genome sequencing capabilities will be critical for enhancing future pandemic responses. SEAR countries should also explore the potential of pathogen-agnostic testing platforms that can be quickly adapted to detect new and emerging infectious diseases, ensuring they remain at the forefront of diagnostic innovation.

**Table 2:** Summary of recommendations to address challenges and constraints to enhance future pandemic preparedness in the South-East Asian region.

Strengthening human resources	Investment in the training and retention of skilled laboratory personnel is critical. Countries should prioritise the development of specialised training programs that can rapidly scale up during emergencies while also ensuring the retention of a stable, well-trained workforce. This includes creating permanent positions for key roles and developing national rosters of trained professionals who can be mobilised in response to surges in demand.
Enhancing laboratory network coordination	Establishing more coherent and integrated laboratory networks is essential for improving the timeliness and equity of public health responses. Countries should focus on developing standardised protocols for specimen referral and transport, ensuring that all regions, including remote and rural areas, have timely access to diagnostic services. Additionally, the establishment of centralised coordination bodies can help streamline laboratory operations and improve communication between national and subnational levels during emergencies.
Securing sustainable funding	Governments in South-East Asia need to develop more robust and flexible funding mechanisms for public health. This includes increasing domestic investment in health systems, establishing emergency funds that can be rapidly mobilised, and fostering public-private partnerships to ensure that resources are available when needed. Sustainable funding is crucial for maintaining laboratory infrastructure, supporting continuous training, and ensuring that countries are prepared to respond effectively to future public health crises.
Improving quality assurance programmes	Developing more flexible and scalable quality assurance frameworks that can be rapidly deployed during public health emergencies is essential. Countries should focus on enhancing quality assurance implementation not only at national levels but also across subnational laboratories to ensure consistent testing reliability. This may involve leveraging technology to conduct remote audits and virtual assessments when in-person inspections are not feasible.
Strengthening biosafety and biosecurity	To address the challenges in biosafety and biosecurity, countries should prioritise the development and implementation of standardised guidelines for laboratory safety. This includes investing in infrastructure upgrades, such as the procurement and certification of biosafety cabinets, and ensuring that laboratories are equipped with the necessary safety equipment. Additionally, the development of more resilient waste management systems is critical to handle the increased demands during a pandemic and to mitigate the risks associated with hazardous waste production.
Enhancing international cooperation	Continued international cooperation is vital for addressing global health challenges. South-East Asian countries should actively participate in international health networks and contribute to global efforts in pandemic preparedness and response. This includes sharing data and research findings, participating in joint training exercises, and collaborating on the development of new diagnostic tools and technologies. By fostering strong international partnerships, countries can better prepare for and respond to future public health emergencies.
Fostering innovation in diagnostic technologies	The pandemic underscored the importance of having access to a wide range of diagnostic tools. Investment in the development of point-of-care testing technologies and the expansion of genome sequencing capabilities will be critical for future preparedness. Countries should also explore the potential of pathogen-agnostic testing platforms that can be rapidly adapted to new and emerging infectious diseases.
Regional Diagnostic Advisory Group	The Regional Diagnostic Advisory Group would enhance ASEAN's diagnostic capabilities by providing expert advice, addressing gaps, and guiding research on new technologies. This group of specialists would prioritize the development of innovative tools, like point-of-care diagnostics, and foster collaboration between governments, researchers, and the private sector. By doing so, it would ensure the timely deployment of effective diagnostic solutions, improving regional preparedness for future health threats.
Genomic surveillance systems	Strengthen genomic surveillance capacity to detect and monitor pathogens with pandemic potential. This includes establishing regional genomic sequencing networks and integrating genomic data into broader public health surveillance systems.

## Conclusions

This report highlights the critical successes and challenges faced by public health laboratories in SEAR during the COVID-19 pandemic, offering key insights into building more resilient and sustainable laboratory systems. Documenting member states' preparedness, interventions, and responses provides valuable lessons for future health emergencies, identifying gaps, optimising laboratory networks, and ensuring infrastructure sustainability. While the region demonstrated strengths in scaling up diagnostic capacities and leveraging international cooperation, the pandemic exposed significant gaps in human resources, coordination, funding, and biosafety. Addressing these challenges requires sustained investment, the development of flexible systems, and strong international partnerships to prepare SEAR for future pandemics and health emergencies.

## Contributors

FYI & PRW–conceptualisation, project administration, funding acquisition, writing–review & editing.

DN, NB, ECS–writing–review & editing.

KKL & SD–writing–original draft, data curation, formal analysis.

SDB–conceptualisation, supervision, methodology, writing–original draft, data curation, formal analysis.

## Declaration of interests

The authors declare no competing interests. The views expressed in the submitted article are solely those of the authors and do not necessarily represent those of their affiliated organisations, including WHO. Nor is an official position of the institution to which they are affiliated.
