# Supplementary material for: Assessment of public health laboratory preparedness and response in WHO South-East Asia region during the COVID-19 pandemic: lessons learned and future directions
Source: Lancet Reg Health Southeast Asia. 2024 Oct 19;31:100496. doi: 10.1016/j.lansea.2024.100496 (PMC11532963; doi:10.1016/j.lansea.2024.100496)
Supplement: Appendix [file mmc1.docx]

**SUPPLEMENTARY APPENDIX 1**

**Assessment of public health laboratory preparedness and response in WHO Southeast Asia region during the COVID-19 pandemic: lessons learned and future directions**

Inbanathan and colleagues; *Lancet Regional Health — Southeast Asia* 2024

[Supplementary Table 1. Methodological details for the Key informant in-depth interviews 2](#_Toc176060071)

[Supplementary Table 2. Standardised questionnaire used in the in-depth interview 3](#_Toc176060072)

[Supplementary Table 3. Study participants and interviewees 5](#_Toc176060073)

[Supplementary Table 4. Scoping review summary results of COVID-19 laboratory responses in WHO South-East Asian Region member states 7](#_Toc176060074)

[References 9](#_Toc176060075)

# Supplementary Table 1. Methodological details for the Key informant in-depth interviews https://www.apple.com/apple-events/ews

|  | Methodological details |
| --- | --- |
| In-depth interviews | Interviews were conducted remotely using internet-based virtual conferencing tools (Microsoft Teams). All interviews were audio- and video-recorded, and participants were informed about the recording. The participants were selected based on discussions with WHO SEA regional and country office staff, with National Laboratory Focal Points (NLFP) as the primary respondents. Tertiary care hospital laboratories or sub-national laboratories from some member states were included based on recommendations from NLFPs. The in-depth interviews focused on technical issues in laboratory systems and did not collect personal clinical or demographic data; thus, no institutional review board review was deemed appropriate. Apart from published literature, country specifics from in-depth interviews were not included in the results or discussions, although participants mentioned in the acknowledgements provided their consent. |
| Data management | Interviewers made detailed notes, which were then consolidated within the research team. All information provided in soft and hard copies was stored following the General Data Protection Regulation (GDPR) requirements. |
| Data analysis | All interviews were transcribed and translated verbatim into English. Transcripts were assessed for accuracy by cross-checking with the audio recordings. Original research questions were used to create a matrix populated by thematic interviewee responses (e.g., pre-pandemic, current, post-pandemic vs. problems, strengths, sustainability, gaps), and the responses were manually analysed. |

# Supplementary Table 2. Standardised questionnaire used in the in-depth interview

| **Introduction** |
| --- |
| 1. Welcome and thank the subject, acknowledgment of time. 2. Introduction – short description of the background of the activity. Note that in this activity, we are interested in people’s experiences relating to the COVID-19 diagnostic preparedness, surge capacity and sustainability issues. |
| **Open conversation** |
| 1. Background of the interviewee    1. What is your position within the organization, and how long have you worked here?    2. How does your position relate to laboratory activities, specifically COVID-19 diagnostics? 2. Pandemic preparedness   4.1 Before January 2020, can you describe pandemic preparedness activities that have been performed for laboratories and can you describe these activities?   - At the national laboratory - For the national laboratory network   4.2 Can you describe the pandemic preparedness activities that have been useful during the COVID-19 pandemic?  4.3 Can you describe what could have been done better regarding pandemic preparedness?   1. COVID-19 diagnostic response   5.1 Can you describe what activities were implemented in response to the COVID-19 pandemic?   - At the national laboratory - For the national laboratory network   *Prompt – ask them to expand on the following areas:*   - Planning - Monitoring and evaluation - Establishing data reporting systems - Communication to subnational laboratories and with public - National Laboratory coordination - Engagement with the private sector  1. COVID-19 diagnostic surge capacity   6.1 Can you describe what laboratory activities were implemented to deal with the increasing number of COVID-19 cases?  6.2 Can you describe what activities implemented as part of the COVID-19 diagnostics surge worked particularly well in the national laboratory  6.3 Can you describe which activities implemented by the national laboratory as part of the COVID-19 diagnostics surge contributed to the laboratory coordinated response at a national level.  6.4 Can you describe the problems experienced with increasing COVID-19 submissions?  *Prompt – if the interview is going well, ask them to expand on the following:*   - Knowledgeable and competent staff - Biosafety and risk mitigation strategies implemented, including waste management - Reusable and disposable personal protective equipment - Diagnostic test to meet the diagnostic workload, including sufficient storage capacity and refrigeration for consumables and samples - Laboratory infrastructure capacity, including electrical supply and maintenance requirements - Budget to perform the mandated activities required of the laboratory during the COVID-19 pandemic - Laboratory information system for reporting laboratory results - Process for national regulation of diagnostics, including local manufacturing capacities  1. COVID-19 diagnostic current situation   7.1 Can you describe what laboratory activities perform well during the current COVID-19 caseload?  7.2 Can you describe what problems you have experienced dealing with the ongoing COVID-19 caseload?  7.3 Can you describe what you think could be done to improve the ongoing situation of COVID-19 diagnostics in your laboratory?  7.4 Can you describe how the laboratory scaled down following peak COVID-19 testing and what lessons were learnt from this process? |
| **Recovery** |
| 1. COVID-19 diagnostic sustainability   8.1 Can you describe your concerns for your laboratory's future?  8.2 Do you think your laboratory will face any unique challenges in the future?  8.3 Can you describe what could be done by donors or international organizations to assist in the future?  8.4 How will you ensure the momentum in diagnostics is maintained for future potential pandemic surge requirements?  8.5 Can you describe which lessons learnt have now been transferred to other diseases /programmes |
| **Wrap up** |
| 1. Close   9.1 Is there anything you would like to add that you think would be important for me to know?  9.2 Is there anything you were surprised I didn’t ask you about?  9.3 Thank the subject for participating in the study and taking the time to talk. |

# Supplementary Table 3. Study participants and interviewees

| **Member state** | **Interviewees** |
| --- | --- |
| Bangladesh | Dr. Zakir Hossain Habib, Chief Scientific Officer, Virology, IEDCR, Mohakhali, Dhaka  Dr. Ahmed Nawsher Alam, Principal Scientific Officer, Virology, IEDCR, Mohakhali, Dhaka  Dr. Manjur Hossain Khan, Assistant Professor, Virology, IEDCR, Mohakhali, Dhaka |
| Bhutan | Dr. Tshokey Tshokey, Clinical Microbiologist, Head of Department, Department of Microbiology, Member of Clinical Advisory Group, National Referral Laboratory  Dr. Sonam Wangchuk, Clinical Microbiologist, Dept of Clinical Microbiology – Head of the Department, Technical advisor and Microbiology Member, Royal Centre for Disease Control |
| India | Dr Nivedita Gupta, Scientist F and Head, Epidemiology and Communicable Diseases, ICMR, New Delhi  Dr Priya Abraham, Director, ICMR-NIV, Pune, India  Dr Pragya Yadav, Scientist F and Group Leader, Maximum Containment Laboratory, ICMR-NIV, Pune, India  Dr Varsha Potdar, Scientist E and Influenza Group Leader, ICMR - NIV, India  Dr. Lata Kapoor, Joint Director, NCDC, MoHFW, New Delhi.  Dr. Monil Singhai, Joint Director, NCDC, MoHFW, New Delhi.  Dr. Nidhi Saini Assistant Director, NCDC, MoHFW, New Delhi. |
| Indonesia | Dr Vivi Setiawaty, Director of Human Resources, Education and Research, National Referral Infectious Disease Hospital, Ministry of Health.  Dr Ir. Anugerah Widiyanto, Director of Human Development, Population, and Culture, National Research and Innovation Agency (BRIN).  Dr Hanief Arief, ST, MAP, Coordinator of Center for Public Health and Community Nutrition, National Research and Innovation Agency (BRIN).  Dr Pretty Multihartina, Head of Center for Health Services, Policy, Ministry of Health.  Dr Muhammad Karyana, Coordinator of Disease Control and Prevention Working Group, Ministry of Health.  Dr Dyah Armi Riana, MARS, Member of Monitoring Working Group, Ministry of Health.  Mr Ferdinan Samson Tarigan, SKM, MKM, Coordinator of Health Promotion of School, Age Child Working Group, Ministry of Health  Dr Herna, Sp.MK, Technical Manager for Polio Laboratory, Ministry of Health.  Dr Harimat Hendrawan, Head of Pre-Clinical and Clinical Medical Research Center, Ministry of Health. |
| Maldives | Dr. Neha Shrestha, Consultant in Microbiology, Republic of Maldives Male, Maldives  Ms. Asifa Luthfy, Senior microbiologist, Republic of Maldives Male, Maldives  Dr. Milza Abdul Muhsin, Pathologist, IGMH, Republic of Maldives Male, Maldives  Ms. Aminath Aroosha, Director Surveillance, HPA, Republic of Maldives Male, Maldives  Ms. Limya Hameed, Assistant Director, QARD, Republic of Maldives, Male, Maldives |
| Nepal | Dr Runa Jha, Director, National Public Health Laboratory  Dr. Rekha Manandhar, Pathology/ Hematology/ Clinical biochemistry unit Head  Ms. Lilee Shrestha, Infectious Disease Unit Head  Mr. Balakrishna Awal, VPD/ HIV/HEP Laboratory Head  Ms. Jyoti Acharya, AMR laboratory Head |
| Sri Lanka | Dr Sudath Dharmaratne, Deputy Director General (Laboratory service), Ministry of Health  Dr Lilani Karunanayake, Consultant Microbiologist, Medical Research Institution  Dr Janaki Abenayake, Consultant Virologist, Medical Research Institution  Dr Sujatha Pathirage, Consultant Microbiologist, Medical Research Institution |
| Thailand | Dr. Archawin Rojanawiwat, Director, Department of Medical Science  Dr. Athiwat Primsirikunawut, Deputy Director, Department of Medical Science  Dr.Pilailuk Okada, Laboratory Manager, Department of Medical Science |
| Timor -Leste | Dr. Ari Jayanti Tilman, Director of Clinical pathology and Microbiology, National Health Laboratory  Mrs. Carolina Maia, Laboratory scientist and AMR focal point, National Health Laboratory  Dr. Rusli Bin Ali, Official Vaccine preventable diseases  Dr. Endang Soares da Silva, Executive Director, National Health Laboratory  Dr Dongbao Yu, WHO Office |

AMR – Antimicrobial resistance

HPA - Health Protection Agency

IEDCR – Institute for Epidemiology Disease Control and Research

ICMR – Indian Council of Medical Research

IGMH – Indira Gandy Memorial Hospital

MoHFW - Ministry of Health and Family Welfare

NCDC - National Centre for Disease Control

QARD – Quality Assurance and Regulation Division

# Supplementary Table 4. Scoping review summary results of COVID-19 laboratory responses in WHO South-East Asian Region member states

| **Member state** | **Pre-Pandemic Preparedness** | **Diagnostic Tests/Capacity** | **Number of COVID-19 testing laboratories** | **Human resources** | **Finance and external cooperation** |
| --- | --- | --- | --- | --- | --- |
| Bangladesh | Previously implemented National Influenza Surveillance, Bangladesh (NISB) and Hospital Based Influenza Surveillance (HBIS) platforms^1^ | SARS-CoV2 rRT-PCR & GeneXpert^®^ ^2^  Rapid antigen tests ^3^ | Centralised at IEDCR in Dhaka^1,4^  118 nationwide ^2^ | Biosafety measures to protect staff ^5^ | SAARC COVID-19 fund ^6^  Multiple Partnerships international health organizations and donor countries ^7^ |
| Bhutan | Pandemic preparedness Influenza ^8^  Simulation exercise for incoming cases of coronavirus ^9^ | SARS-CoV2 rRT-PCR, High testing rate per capita ^10^ | Royal Center for Disease Control (RCDC) expanded to five centres across the country ^11^ | Limited staff due to geography ^11^ | WHO ^9^ |
| DPR Korea | Reported investments in healthcare system ^12^ | Purported local SARS-CoV2 rRT-PCR development ^13^ | NA | NA | WHO ^14^ |
| India | WHO SEAR regional influenza pandemic preparedness plan (2006-2008) ^15^ | SARS-CoV2 rRT-PCR, Rapid antigen tests  ^16,17^ | Extensive network ^18^  Over 1,596 by August 2020 ^19^ | Biosafety measures to protect staff ^20^ | WHO ^19^ |
| Indonesia | National pandemic preparedness plan for avian influenza and SARS ^21,22^ | SARS-CoV2 rRT-PCR ^23,24^  February 2021 - monthly average of specimens tested was 56,015 and people tested was 37,519 ^25^ | 685 labs across 34 provinces ^24,25^  558 diagnostic laboratories equipped with RT-PCR, 70 with molecular rapid test (TCM) and 57 with RT-PCR and TCM equipment ^23,24^ | COVID-19 biosafety measures to protect staff ^5^ | WHO and European Union ^26^ |
| Maldives | WHO SEAR regional influenza pandemic preparedness plan (2006-2008) ^15^ | SARS-CoV2 rRT-PCR^27-30^  GeneXpert^® 30^  Rapid antigen tests^30^ | Private & Government laboratories^31-33^ | COVID-19 diagnostics training programs for laboratory staff ^30^ | WHO ^30,32,34^ |
| Myanmar | WHO SEAR regional influenza pandemic preparedness plan (2006-2008) ^15^ | SARS-CoV2 rRT-PCR ^28,29^  Rapid antigen tests ^29,35^ | 6 labs (Yangon, Nay Pyi Taw, Mandalay, Mawlamyine) ^29^ | 8 staff working two shifts ^29^ | WHO, UNOPS & UNICEF ^36^  UNHCR, WFP ^37^ |
| Nepal | WHO SEAR regional influenza pandemic preparedness plan (2006-2008) ^15^ | SARS-CoV2 rRT-PCR ^38^ | 82 labs by late 2020 ^38^ | Workforce development requirement noted as part of preparedness ^15^ | SAARC COVID-19 fund ^6^ |
| Sri Lanka | WHO SEAR regional influenza pandemic preparedness plan (2006-2008) ^15^ | SARS-CoV2 rRT-PCR ^39^  MoH capacity 1200 PCR tests/day ^39^ | Multiple laboratories – Development of national laboratory capacity noted ^39^ | Workforce development requirement noted as part of preparedness ^15^  COVID-19 biosafety measures to protect staff ^40^ | WHO ^39^ |
| Thailand | WHO SEAR regional influenza pandemic preparedness plan (2006-2008) ^15^ | SARS-CoV2 rRT-PCR &  Rapid antigen tests ^41^ | Existing rapid laboratory support system ^35^  More than 1800 laboratories ^42^ | Workforce development noted during the pandemic ^42^ | Laboratory costs covered by the Department of Disease Control or public insurance schemes ^35^ |
| Timor Leste | Molecular Diagnostic Laboratory at NHL (NHL- MDL), Timor-Leste was established in 2011 as part of National Pandemic Influenza Preparedness (PIP) laboratory response ^43^ | SARS-CoV2 rRT-PCR^43,44^ & GeneXpert^®^ ^43^  Established molecular testing service ^43^  Between March 2020 and February 2022, conducted over 200,000 molecular tests ^43^ | Laboratories established in all 13 provinces ^43^  Up to 2000 tests performed daily ^44^ | Increased from 5 to 28 scientists ^43^ | Fleming Fund Grant (UKAid)  UN Organisations (WHO-TL, UNDP, UNICEF, and IOM), foreign embassies and associated organisations (DFAT, Menzies, USAID, KOICA) ^43,44^ |

NA – Not available

IEDCR - Institute of Epidemiology Disease Control and Research - Bangladesh

NHL - National Health Laboratory (NHL) – Timor-Leste

DFAT - Australian Government Department of Foreign Affairs and Trade

KOICA - Korea International Cooperation Agency

WHO-TL – World Health Organisation – Timor Leste

Menzies - Menzies School of Health Research, Charles Darwin University, Darwin, Australia

UNDP – United Nations Development Program

IOM – International organisation for migration

UNICEF – United Nations Children’s Fund

UNHCR – United Nations high commission for refugees

WFP – World Food Program

UNOPS – United Nations Office for Program Services

# References

1. Tabassum T, Farzana M, Nahar AN, et al. COVID-19 in Bangladesh: Wave-centric assessments and mitigation measures for future pandemics. *Heliyon* 2023; **9**(10): e20113.

2. World Health Organisation. Get tested! WHO supports the Government of Bangladesh in establishing a broad testing lab network throughout the country. 2021. <https://www.who.int/bangladesh/news/detail/30-03-2021-get-tested!-who-supports-the-government-of-bangladesh-in-establishing-a-broad-testing-lab-network-throughout-the-country> (accessed 27 April 2024).

3. Sania A, Alam AN, Alamgir ASM, et al. Rapid antigen testing by community health workers for detection of SARS-CoV-2 in Dhaka, Bangladesh: a cross-sectional study. *BMJ Open* 2022; **12**(6): e060832.

4. Government of the People’s Republic of Bangladesh. Bangladesh Preparedness and Response Plan for COVID-19. 2020. <https://www.humanitarianresponse.info/sites/www.humanitarianresponse.info/files/documents/files/nprp_covid-19_v6_18032020.pdf> (accessed 2 April 2023).

5. Islam SRU, Akther T, Sultana S, et al. Challenges in the establishment of a biosafety testing laboratory for COVID-19 in Bangladesh. *J Infect Dev Ctries* 2021; **15**(12): 1833-7.

6. Pattanaik SS. SAARC COVID-19 Fund: Calibrating a Regional Response to the Pandemic. *Strategic Analysis* 2020; **44**(3): 241–52.

7. Mohiuddin AK. COVID-19 and 20 Resolutions for Bangladesh. *European Journal of Sustainable Development Research* 2020; **4**(4): em0139.

8. Ministry of Health - Bhutan. Operational Guideline For Influenza-Like Illness and Severe Acute Respiratory Infection. 2014. <http://www.rcdc.gov.bt/web/wp-content/uploads/2022/07/ILI-SARI_gudeline-2nd-edition.pdf>.

9. World Health Organization. “Invest in preparedness” – Health emergency readiness lessons from Bhutan. 2020. <https://www.who.int/news-room/feature-stories/detail/invest-in-preparedness-health-emergency-readiness-lessons-from-bhutan> (accessed 26 March 2023).

10. Dorji T. The Gross National Happiness Framework and the Health System Response to the COVID-19 Pandemic in Bhutan. *Am J Trop Med Hyg* 2021; **104**(2): 441-5.

11. Yangchen S, Ha S, Assan A, Tobgay T. Factors influencing COVID-19 testing: a qualitative study in Bhutan. *Glob Health Res Policy* 2022; **7**(1): 10.

12. Park K, Ham E. North Korea’s Surprisingly Robust Healthcare System. 2021. <https://www.globalasia.org/v16no3/cover/north-koreas-surprisingly-robust-healthcare-system_kee-b-parkedward-i-ham> (accessed 26 March 2023).

13. Shin H. N. Korea develops real-time PCR equipment to test for coronavirus: state media. 2021. <https://www.reuters.com/world/asia-pacific/nkorea-develops-its-own-pcr-equipment-covid-19-tests-state-media-2021-08-23/> (accessed 23 March 2023).

14. World Health Organisation. WHO committed to support DPR Korea respond to COVID-19 pandemic. 2022. <https://www.who.int/southeastasia/news/detail/16-05-2022-WHO-committed-support-DPR-Korea-respond-to-covid-19-pandemic> (accessed 3 May 2024).

15. World Health Organisation. Regional Influenza Pandemic Preparedness Plan (2006-2008). 2006 <https://iris.who.int/bitstream/handle/10665/205803/B0167.pdf> (accessed 29 April 2024).

16. Gupta N, Bhatnagar T, Rade K, et al. Strategic planning to augment the testing capacity for COVID-19 in India. *Indian J Med Res* 2020; **151**(2 & 3): 210-5.

17. Kumar KSR, Mufti SS, Sarathy V, Hazarika D, Naik R. An Update on Advances in COVID-19 Laboratory Diagnosis and Testing Guidelines in India. *Front Public Health* 2021; **9**: 568603.

18. Abraham P, Aggarwal N, Babu G, et al. Laboratory surveillance for SARS-CoV-2 in India: Performance of testing & descriptive epidemiology of detected COVID-19, January 22 - April 30, 2020. *Indian J Med Res* 2020; **151**(5): 424-37.

19. World Health Organisation. How India scaled up its laboratory testing capacity for COVID19. 2020. <https://www.who.int/india/news/feature-stories/detail/how-india-scaled-up-its-laboratory-testing-capacity-for-covid19> (accessed 3 May 2024).

20. Mourya DT, Sapkal G, Yadav PD, SK MB, Shete A, Gupta N. Biorisk assessment for infrastructure & biosafety requirements for the laboratories providing coronavirus SARS-CoV-2/(COVID-19) diagnosis. *Indian J Med Res* 2020; **151**(2 & 3): 172-6.

21. Hanvoravongchai P, Adisasmito W, Chau PN, et al. Pandemic influenza preparedness and health systems challenges in Asia: results from rapid analyses in 6 Asian countries. *BMC Public Health* 2010; **10**: 322.

22. Setiawaty V, Pangesti KNA, Sampurno OD. Establishing a laboratory network of influenza diagnosis in Indonesia: An experience from the avian flu (H5N1) outbreak. *Clinical Epidemiology* 2012; **4**(1): 209-12.

23. Hendarwan H, Syachroni S, Aryastami NK, et al. Assessing the COVID-19 diagnostic laboratory capacity in Indonesia in the early phase of the pandemic. *WHO South East Asia J Public Health* 2020; **9**(2): 134-40.

24. Aisyah DN, Mayadewi CA, Budiharsana M, et al. Building on health security capacities in Indonesia: Lessons learned from the COVID-19 pandemic responses and challenges. *Zoonoses Public Health* 2022; **69**(6): 757-67.

25. Aisyah DN, Mayadewi CA, Igusti G, Manikam L, Adisasmito W, Kozlakidis Z. Laboratory Readiness and Response for SARS-Cov-2 in Indonesia. *Front Public Health* 2021; **9**: 705031.

26. World Health Organisation. WHO and European Union strengthen Indonesia’s health system for a healthier tomorrow. 2024. <https://www.who.int/indonesia/news/detail/19-02-2024-who-and-european-union-strengthen-indonesia-s-health-system-for-a-healthier-tomorrow> (accessed 29 April 2024).

27. Sarkar A, Liu G, Jin Y, Xie Z, Zheng ZJ. Public health preparedness and responses to the coronavirus disease 2019 (COVID-19) pandemic in South Asia: a situation and policy analysis. *Glob Health J* 2020; **4**(4): 121-32.

28. Phyu WW, Saito R, Wagatsuma K, et al. Epidemiology and Genetic Analysis of SARS-CoV-2 in Myanmar during the Community Outbreaks in 2020. *Viruses* 2022; **14**(2).

29. Frontier. Testing times: Myanmar struggles to keep pace as COVID-19 takes off. 2020. <https://www.frontiermyanmar.net/en/testing-times-myanmar-struggles-to-keep-pace-as-covid-19-takes-off/> (accessed 28 April 2024).

30. World Health Organisation. COVID-19 Preparedness and Response Plan Maldives. 2020. <https://maldives.un.org/en/102366-covid-19-preparedness-and-response-plan-maldives> (accessed 29 April 2024).

31. World Health Organisation. WHO Helps Maldives Build Laboratory Capacity to test COVID-19. 2021. <https://maldives.un.org/en/116412-who-helps-maldives-build-laboratory-capacity-test-covid-19> (accessed 28 April 2024).

32. World Health Organisation. Health system response to Covid-19, Republic of Maldives: World Health Organisation, 2020.

33. Pooransingh S, Yoosuf AA, Moosa S, Ahmed N, Jankie S, Pinto Pereira L. Early COVID-19 response in two small island developing states: Maldives and Trinidad and Tobago. *Western Pac Surveill Response J* 2022; **13**(1): 1-7.

34. World Health Organization. WHO Helps Maldives Build Laboratory Capacity to test COVID-19. 2020. <https://www.who.int/southeastasia/news/feature-stories/detail/who-helps-maldives-build-laboratory-capacity-to-test-covid-19> (accessed 20 May 2023).

35. Rampal L, Liew BS, Choolani M, et al. Battling COVID-19 pandemic waves in six South-East Asian countries: A real-time consensus review. *Med J Malaysia* 2020; **75**(6): 613-25.

36. Ranoev V. UN Supplies Over 75,000 Test Kits to Government of Myanmar in Fight Against COVID-19. 2020. <https://myanmar.un.org/en/49025-un-supplies-over-75000-test-kits-government-myanmar-fight-against-covid-19> (accessed 28 April 2024).

37. Linn HO. UNHCR and WFP team up to deliver 20,000 COVID-19 Test Kits to Myanmar. 2020. <https://myanmar.un.org/en/78410-unhcr-and-wfp-team-deliver-20000-covid-19-test-kits-myanmar> (accessed 28 April 2024).

38. Khadka RB, Gyawali R. Establishing PCR Testing in Nepal for Covid-19: Challenges and Opportunities. *Kathmandu Univ Med J (KUMJ)* 2020; **18**(71): 309-12.

39. Ministry of Health and Indigenous Medical Services Sri Lanka. Sri Lanka Preparedness & Response Plan, COVID-192020. <http://www.health.gov.lk/moh_final/english/public/elfinder/files/news/2020/FinalSPRP.pdf> (accessed.

40. Munasinghe NL, O'Reilly G, Cameron P. Lessons learned from the COVID-19 response in Sri Lankan hospitals: an interview of frontline healthcare professionals. *Front Public Health* 2023; **11**: 1280055.

41. World Health Organization. Joint Intra-Action Review of the Public Health Response to COVID-19 in Thailand. 2020. <https://www.who.int/docs/default-source/searo/thailand/iar-covid19-en.pdf> (accessed 14 April 2024).

42. Goel V, Mathew S, Gudi N, Jacob A, John O. A scoping review on laboratory surveillance in the WHO Southeast Asia Region: Past, present and the future. *J Glob Health* 2023; **13**: 04028.

43. Sarmento N, Soares da Silva E, Barreto I, et al. The COVID-19 laboratory response in Timor-Leste; a story of collaboration. *Lancet Reg Health Southeast Asia* 2023; **11**: 100150.

44. Francis JR, de Araujo RM, da Silva Viegas O, et al. The response to COVID-19 in Timor-Leste: lessons learnt. *BMJ Glob Health* 2023; **8**(10).
